# Screening for Tuberculosis and Its Histological Pattern in Patients with Enlarged Lymph Node

**DOI:** 10.4061/2011/417635

**Published:** 2011-05-12

**Authors:** Hussain Gad ElKarim Ahmed, Akram Saleh Nassar, Ibrahim Ginawi

**Affiliations:** ^1^Department of Histopathology and Cytology, Faculty of Medical Laboratory Sciences, University of Khartoum, Khartoum, Sudan; ^2^National Tuberculosis Center, Sana'a, Yemen; ^3^College of Medicine, University of Ha'il, Ha'il, Saudi Arabia

## Abstract

*Settings*. Tuberculosis is a major health problem in the Republic of Yemen. Tuberculous lymphadenitis is one of the most common forms of extrapulmonary tuberculosis. Therefore, this retrospective descriptive study was conducted in Yemen to investigate the morphological pattern of tuberculous lymphadenitis, as well as to assess the reliability measures of (ZN) Ziehl-Neelsen and fluorescent methods in identification of *Mycobacterium tuberculosis*. *Methodology*. One hundred lymph nodes tissue biopsies that were previously diagnosed by conventional histopathology as having tuberculous lymphadenitis were reinvestigated. Five micron in thickness sections were obtained from formalin-fixed paraffin wax processed tissues. The sections were stained using Haematoxylin and eosin (H & E), ZN, and fluorescent methods. *Results*. All of the 100 specimens were proved as having histopathological pattern of tuberculosis lymphadenitis. The most major histological features were giant cell (88%), caseation (84%), epithelioid cells (80%), granuloma and caseation (68%), lymphocytes (31%), and histiocytes (4%). After staining the specimens with ZN and fluorescent, of the 100 specimens only 3 (3%) and 9 (9%) specimens were found positive, by ZN and fluorescent methods, respectively. *Conclusion*. Conventional ZN and fluorescent methods have limitations in diagnosis of tuberculous lymphadenitis due to their lower sensitivity. Histopathology remains the most suitable method for the diagnosis of tuberculous lymphadenitis. In cases of suspected tuberculous lymphadenitis, it is advisable to confirm with more sensitive and specific method, such as polymerase chain reaction PCR or immunohistochemistry before reporting the negative results.

## 1. Introduction

Tuberculosis (TB) of the lymph node (tuberculosis lymphadenitis) is the most common form of extrapulmonary tuberculosis. In developing countries where the incidence of TB is high, tuberculosis lymphadenitis is one of the most frequent causes of lymphadenopathy (30–52%) [1].

TB is among the foremost infectious causes of mortality worldwide, with 2–3 million deaths reported each year. Tuberculosis causes the second highest mortality rates from an infectious disease worldwide, after human immunodeficiency virus. Currently, 30% of the world's population is estimated to be infected with the disease [2]. 

In Yemen, tuberculosis is considered as one of the most common causes of death. The absolute number of cases in Yemen is not known, but about 37000 cases were under treatment in all over the country for the year 2002 [3]. The main risk factors for tuberculosis include older age, lower socioeconomic status (via crowding, homelessness, poor nutrition, etc.), and HIV [4]. Other risk factors for the development of tuberculosis include smoking, alcohol consumption, shortage of food, and contact with TB patients [5]. 

Diagnosis of tuberculous lymphadenitis still faces many challenges, though there are many applied diagnostic tools. The diagnosis of extrapulmonary TB is difficult, especially when clinical presentation is suggestive but bacteriological proof is lacking. The diagnosis confirmed by acid-fast bacilli (AFB) using conventional microscopy is simple and rapid but lacks sensitivity whereas culture is more sensitive and specific but takes several weeks to get the results [6]. However, early diagnosis of infection is important before the use of antituberculosis chemotherapy. Clinical diagnosis is usually dependent on microscopic detection of *M. tuberculosis* using Ziehl-Neelsen stain and mycobacterium culture [7]. Fluorescent stain (FS) has been proven to be superior to the Ziehl-Neelsen (ZN) stain, especially in paucibacillary cases [8, 9]. Fine needle aspiration cytology is a quick, minimally invasive, and cost-effective technique for the diagnosis of granulomatous diseases [10]. 

The PCR is a sensitive and specific technique which is frequently introduced in the diagnosis of tuberculous lymphadenitis. Tests employing polymerase chain reaction for the specific detection of mycobacterium belonging to the *M. tuberculosis* complex [11]. Multiplication of tubercle bacilli in any site of the human body causes a specific type of inflammation, with formation of a characteristic granuloma [12].

Tuberculosis is still a public health problem in Yemen and is one of the ten major causes of death. Although, tuberculous lymphadenitis is one of the most common forms of extrapulmonary tuberculosis, very few studies on the histopathology of this condition have been done in Yemen.

 Therefore, the purpose of the current study was to study the common histopathological changes of lymph nodal tuberculosis and to assess the possibility of identifying the *M. tuberculosis* in histological section using ZN and fluorescence techniques.

## 2. Materials and Methods

**Figure 1:**
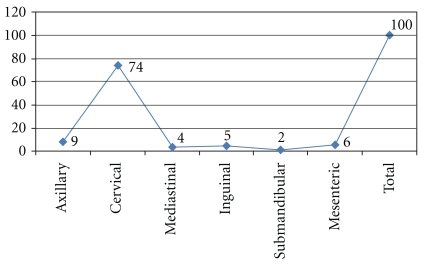
Showing description of the study population by lymph node site.

This is a descriptive study to screen patients with enlarged lymph node for *Mycobacterium tuberculosis.* The study was conducted in Sana'a, Yemen during the period from 2007 to 2009. One hundred lymph node biopsies were retrieved from 631 lymph node biopsies, which were previously obtained from patients with enlarged lymph nodes. The biopsies' sites were shown in Figure 1. All specimens were formalin-fixed paraffin wax processed tissues. Information regarding each patient was obtained from each patient's file. The specimens were fixed in 10% formalin and then processed by tissue processing machine using the following schedule adopting 24-hour scheduling. Three 5-microns thickness sections were obtained from each patient's block using Rotary Microtome. Of the 3 sections, each one was stained with one staining procedure (haematoxylin and eosin, ZN, or fluorescence).

### 2.1. Data Analysis

Data were analyzed using a computer SPSS program (version 12).

## 3. Results

In this descriptive study, we assessed the histopathological pattern of TB in 100 tuberculosis patients, their ages ranging from 7 to 86 years with a mean age of 29 years old, as indicated in Table 1. The male female ratio was 1 : 2.3.

Table 2 is showing the distribution of the study subjects by lymph node site and TB results. The great majority of the specimens were obtained from cervical lymph node followed by axillary lymph node representing 74 (74%) and 9 (9%), respectively. The remaining sites include mesenteric, inguinal, mediastinal, and submandibular, constituting 6 (6%), 5 (5%), 4 (4%), and 2 (2%) correspondingly. 

In this study, 100 patients with enlarged lymph nodes were diagnosed as having lymph node tuberculosis by histopathology. These patients were further divided into two groups according to the presences of strong and weak tuberculosis histopathological evidences. 

Those showing histopathological pattern containing (giant cells + granuloma + caseation) were considered as strong evidence, and the other showing less evidences (e.g., ill-defined aggregates of epithelioid histiocytes only, palisading granulomas without necrosis and giants cells, etc.) were considered as weaker evidence. Accordingly, the strong evidence (positive) was used as a gold standard for comparing the other variables. Accordingly, of the 100 patients, 68 were categorized as having strong evidences (positive) and the remaining 32 were detected with weaker evidences (positive), cases. 

Of the 100 studied lymph nodes, only 3 (3%) were demonstrated as positive in ZN (in all cases more than 5 bacilli were seen). The entire 3 ZN positive were previously found as strong evidence positive.

On staining of the lymph node by fluorescent method, 9 (9%) were found positive for *M. tuberculosis*. Out of the 9 positive, 7 (7%) were identified as strong positive and the remaining two were at negative level. 

## 4. Discussions

In this study, all of the study subjects (*n* = 100) were previously diagnosed as having lymph node TB, depending on the presences of variable histological TB evidences. To strength our findings, we reassessed these evidences, by grouping of the histological features, depending on the strongest histological features of TB. All histological tissues that showed the presence of caseation, granuloma, and Langhans giant cells were considered as positive “strong standard.” Out of the 100 TB patients, 68 (68%) were found with strong histological evidence of TB. Such findings have been previously reported, separately counting different features [4]. For histopathology, the sections were examined for the presence of granuloma and subdivided into two groups for analysis. Well-organized granulomas were characterized by a central group of epitheloid histiocytes, Langhan's giant cells, a mantle of lymphocytes and fibrous tissue. Poorly organized granulomas showed a diffuse mixture of lymphocytes, histiocytes, and plasma cells with occasional giant cells. Each granuloma was also analyzed for the presence or absence of necrosis. The number of granulomas per a sections, their type of organization, and presence of necrosis were noted [13]. There are two specific pathologic criter for identifying tuberculosis lymphadenitis, caseation and granuloma formation. Caseation has been found to be more specific and sensitive [14]. In the present study, 68 lymph nodes were of caseating granuloma type and 32 were of noncaseating granuloma type. This is in correlation with [15], who documented 67% caseating granuloma. Kumar et al. [8] Lake and Oski [9] reported 76% caseating granuloma and 24% noncaseating granuloma, and Fatmi and Jamal [16] reported 62% caseating granulomas and 38% non caseating granuloma. 

In this study, of the 100 TB patients only 3% and 9% were found to be TB positive with ZN and fluorescence stains, respectively. Conventional diagnostic tests for tuberculosis have several limitations and are often unhelpful in establishing the diagnosis of extrapulmonary tuberculosis. Acid-fast bacilli (AFB) positivity in smears and histological specimens depends on the bacillary load of the specimen and the type of the material [17]. Different studies have reported a wide range of AFB positivity ranging from as low as 0% to as high as 75% [18]. It was reported that absence of AFB in samples showing an otherwise characteristic histopathological picture should not weigh against the diagnosis of tuberculosis [19–21]. Fluorescent stain was found to be the most sensitive (81.8%) of the conventional methods but showed poor specificity (28.2%) [22]. The most common site of tuberculosis lymphadenitis is in the neck along the sternocleidomastoid muscle. It is usually unilateral and causes little or no pain. Advanced cases of tuberculosis lymphadenitis may suppurate and form a draining sinus. In the current study, the great majority of the specimens were obtained from cervical lymph node (74%), but less frequently than in other studies in which cervical nodes were affected in about 90% of cases [23]. Our results showed that tuberculosis lymph nodes can infect adults and children at any time and age in which the disease appeared among the patients at ages starting from 7 to 86 years, but the statistical results showed that the patients' ages ranged 7–22 and 23–38 years old, respectively, are the patients that most harbor the disease. These results are in agreement with the study by Nomani et al. [24] they showed that the maximum incidence was found to be in the age group 10–30 years. In the United States, more than 60% of TB cases occur in persons aged 25–64 years; however, the age-specific risk is highest in persons older than 65 years [25]. TB infection in infants and young children (≤5 y) always indicates recent transmission. However, in this study, the rate of infection among females was 70% which is higher than infection among males which was 30%. These results were in agreement with another study by Fatmi and Jamal [16], they reported that out of (100) lymph node tuberculosis 66 patients (66%) were females and 34 (34%) were males.

 In conclusion, although pulmonary TB represents a major health problem, tuberculosis lymphadenitis represents another extra burden in Yemen. Conventional ZN and fluoresces methods for tuberculosis have several limitations and are often unhelpful in establishing the diagnosis of lymph node tuberculosis. Histopathology remains one of the most important methods for diagnosing tuberculosis, and in a high TB prevalent area histopathology is the reliable and a gold standard (as otherwise is the culture). However, it cannot differentiate changes caused by *M. tuberculosis*, *nontuberculous mycobacterium*, or other granulomatous diseases. In endemic countries, the majority of granulomatous lesions without necrosis are considered to be tuberculosis, but this may not be the case in the developed world. Therefore, it is advisable to use more specific and sensitive methods, such as, PCR or immunohistochemistry, before reporting the negative results.

## Figures and Tables

**Table 1:** Distribution of the study population by age.

Age∖years	Frequency	Percent	Cumulative percent
7–22	44	44.0	44.0
23–38	35	35.0	79.0
39–54	13	13.0	92.0
55–69	6	6.0	98.0
70–89	2	2.0	100.0

Total	100	100.0	

**Table 2:** Distribution of the study subjects by lymph node site and TB strong or weak evidences.

Site	TB evidence		Total
	Strong	Weaker	
Axillary	2	7	9
Cervical	22	52	74
Mediastinal	1	3	4
Inguinal	4	1	5
Submandibular	1	1	2
Mesenteric	2	4	6

Total	32	68	100
